# A novel weevil-transmitted tymovirus found in mixed infection on hollyhock

**DOI:** 10.1186/s12985-023-01976-6

**Published:** 2023-01-30

**Authors:** Mathieu Mahillon, Justine Brodard, Isabelle Kellenberger, Arnaud G. Blouin, Olivier Schumpp

**Affiliations:** Research Group Virology, Bacteriology and Phytoplasmology, Agroscope, Nyon, Switzerland

**Keywords:** Tymovirus, Tymoviridae, Weevil, Hollyhock, Mixed infection, Carlavirus, Potyvirus

## Abstract

Leaves of hollyhock (*Alcea rosea*) exhibiting vein chlorosis and yellow mosaic symptoms were collected at public sites in Lausanne and Nyon, two cities of western Switzerland. Diagnostic methods untangled in samples from both sites the mixed infections of a novel isometric virus, tentatively named “Alcea yellow mosaic virus” (AYMV) with the carlavirus Gaillardia latent virus. A new potyvirus was also identified in samples from Nyon. A combination of Illumina, Nanopore and Sanger sequencing was necessary to assemble the full-length genome of AYMV, revealing an exceptionally high cytidine content and other features typically associated with members of the genus *Tymovirus*. The host range of AYMV was found to be restricted to mallows, including ornamentals as well as economically important plants. Phylogenetic analyses further showed that AYMV belongs to a *Tymovirus* subclade that also gathers the other mallow-infecting members. The virus was readily transmitted by sap inoculation, and the weevil species *Aspidapion radiolus* was evidenced as a vector. Transmission assays using another weevil or other insect species did not succeed, and seed transmission was not observed.

**Supplementary Information:**

The online version contains supplementary material available at 10.1186/s12985-023-01976-6.

## Introduction

The genus *Tymovirus* (*Tymovirales*: *Tymoviridae*) accommodates isometric viruses infecting dicotyledons [1]. Members of this taxon have historically served as models for studies of virion structure, transfer RNA (tRNA) mimicry and RNA silencing [2–5]. Several members are also pathogens of economically important crops. For instance, the type member turnip yellow mosaic virus (TYMV) infects diverse *Brassica* plants in Eurasia and Australia [6, 7]. Another example is tomato blistering mosaic virus (TBMV), an emerging threat to tomato and tobacco production in South America [8, 9]. In parallel, some members have been identified on non-crop plants, revealing interesting aspects of viral ecology in wild natural habitats [10–12]. Tymoviruses are often found at high titer in plant tissues and typically induce yellow mosaic symptoms on leaves. Transmission can be mediated via chewing insects from the order *Coleoptera* [1], plant-to-plant contact, agricultural machineries, and in rare cases through seeds [13, 14].

The genomic RNA (gRNA) of tymoviruses is a positive-sense, single-stranded RNA (+ ssRNA) that ranges from 6035 to 6679 nt in length [1]. It harbors a 5’ cap and ends by a tRNA-like structure (TLS) with a few exceptions [15, 16]. Most molecular data regarding the tymoviruses have been collected from TYMV for which the model species *Arabidopsis thaliana* is a suitable host [17]. Three ORFs are present on the TYMV genome; two overlapping ORFs encoding for the movement protein (MP) and the replication protein (RP) followed by small ORF encoding for the capsid protein (CP) [1]. The MP acts as a viral suppressor of RNA silencing (VSR) [18] and is involved in symptom induction [19]. The RP, translated by leaky scanning, is a polyprotein displaying activities of methyltransferase (Met), papain-like protease (Pro), helicase (Hel) and RNA-dependent RNA polymerase (RdRp). Following two Pro-mediated cleavages, the RP produces three mature peptides that enable replication in chloroplast-associated vesicles [20, 21]. The CP ORF is translated from a subgenomic RNA (sgRNA) for which the promoter is a conserved sequence named the “tymobox” [22].

The family *Malvaceae*, commonly referred to as the mallows, is a large group of flowering plants which comprises several species of economic importance. To date, two tymoviruses have been retrieved from mallows, namely okra mosaic virus (OMV) and cacao yellow mosaic virus (CYMV), both present in Sub-Saharan Africa [23–25]. The mallows also include numerous ornamentals such as the hollyhock, a popular biennial plant commonly grown in private gardens and public areas. In this study, we present a novel tymovirus retrieved from symptomatic hollyhocks in two cities of western Switzerland.

## Material and method

### Symptomatic plant material

Leaf samples from symptomatic hollyhocks were collected in two green parks in Lausanne (46°31′10.9" N 6°35′57.1"E) and Nyon (46°23′05.4" N 6°14′06.4" E) in May 2021 and were stored at 4 °C until further use.

### Mechanical inoculation

The leaf material was ground in cold phosphate buffer (20 mM Na_2_HPO_4_, pH 7.6, supplemented with 1 mM diethyldithiocarbamate) in a mortar using a pestle. The sap was then rubbed on the first true leaves of tested plants using carborundum (400-mesh silicon carbide, Aldrich) as abrasive. Plants were inoculated when the first leaves were fully developed and then maintained for at least two months in greenhouse conditions (24 °C, 14/10 h photoperiod) with daily inspection for symptom expression. Inoculation of semi-purified particles was performed in a similar way.

### Semi-purification of virions

A protocol developed for nepoviral particles was followed with some modifications [26]. Briefly, leaves were first ground in liquid N_2_ using a mortar and a pestle. The obtained powder was mixed with a cold extraction buffer (17 mM citric acid, 167 mM NaHPO_4_, 0.2% thioglycolic acid, pH 7.0) in a ratio 1:4 (w/v). The solution was slowly mixed on ice for 20 min and then filtered twice through cheesecloth. The filtrate was next clarified with 0.5 vol chloroform and 0.5 vol butanol. After slow mixing on ice for 20 min, the solution was centrifuged at 4000 rpm for 20 min. The supernatant was collected and centrifuged for 1 h at 40,000 rpm. The obtained pellet was resuspended in 1:10 diluted extraction buffer. Semi-purified particles were denatured in Laemmli buffer and then analysed by SDS-PAGE using a precast 12% acrylamide gel (Bio-Rad). An unstained, SDS-PAGE standards low range ladder (Bio-Rad) was used for size estimation. The gel was stained with Coomassie brilliant blue R 250 (Fluka). Total RNA was extracted from semi-purified particles via the RNeasy plant kit (Qiagen, with DNAse I treatment), and submitted to an agarose gel electrophoresis. For this gel as well as other agarose gels, a 100 bp DNA ladder was used.

### Transmission electron microscopy

Leaf crude extracts and semi-purified particles were analyzed by transmission electron microscopy (TEM). Samples were first mixed with one vol of 0.1% bovine serum albumin and one vol of 4% phosphotungstic acid (pH 6.0). One drop of the resulting solution was sprayed onto Formvar/Carbon 400-Mesh copper grids (Agar Scientific) using a homemade device. Grids were then observed with the Tecnai G2 Spirit electron microscope (FEI).

### RT-PCR and Sanger sequencing

RT-PCR analyses were performed on CTAB-extracted nucleic acids as previously described [27] using the primers listed in Table 1. When necessary, amplicons were cloned in the pGEM-T plasmid with T4 DNA ligase (Promega). Recombinant plasmids were then used to transform *E. coli* DH5alpha and subsequently sequenced by Fasteris (Switzerland) or Microsynth (Switzerland) using M13 universal primers.

**Table 1:** Primers used in this study

Name	Sequence	Use	References
CIFor	GGIVVIGTIGGIWSIGGIAARTCIAC	Potyvirus generic detection	[28]
CIRev	ACICCRTTYTCDATDATRTTIGTIGC		
TymZ-F	GGSCCMGTSAARAARTAYCA	Tymovirus generic detection	[29]
TymZ-R	GCCAGRTTGTARTCRGRGTTG		
CarF2b	GGRCTDGGDGTVCCNACTGA	Carlavirus generic detection	[30]
CarR	CCWCATYSRCTCSRCTWTGG		
TymoFinalF	CCCTACACCACGTCCCATTC	AYMV assembly gap & detection	This study
TymoFinalR	CAAGTGGGGTCGGTCAGAAG		
Seq5two	GATGGCCCAGGGAAGTTCTG	AYMV 5’ terminus	
Tymo5closer	TCCGTGACCATCTCTGGAGT	AYMV 3’ terminus	
AnchoredPolyA	A_22_BNN		
Carla_GLV_For	GGTACAGGAATTGGCTGGCT	GLV detection	
Carla_GLV_Rev	TTCCAATACTGACGGCTCGG		
Poty_Malva_Fwd	GAACCAACACGACCACTTGC	Novel potyvirus detection	
Poty_Malva_Rv	AGTGTGGGTTGGCTGAAGTT		

I = inosine, Y = C/T, B = C/G/T, R = G/A, W = A/T, V = A/C/G, S = C/G and D = A/G/T

### Extraction of dsRNA

A cellulose-based protocol was followed to extract the viral double-stranded RNA (dsRNA) from infected leaves [31]. The dsRNA was resuspended in water and then treated with DNase QI (Qiagen) and S1 nuclease (Promega) to remove plant DNA and RNA.

### Genome sequencing

For the full-length genome sequencing, the tymovirus isolated from Lausanne was used. The dsRNA extracted from infected *M. sylvestris* plants was first quantified using the Qubit 3 Fluorometer (Invitrogen), and then sequenced by high-throughput sequencing (HTS) on a Illumina platform at Macrogen (South Korea), using a paired-end, 100 bp-read cDNA library. A total of 32,314,372 reads were generated, of which > 95.2% exhibited good quality scores (> Q30). Reads were trimmed with Trimmomatic [32] and assembled using Spades [33]. Viral contigs were then sorted based on Blastx and Blastn searches in the NCBI databases limited to the taxid “virus”. Gaps between viral contigs were filled by RT-PCR and subsequent Sanger sequencing, except for one region that was sequenced on a MinION Flongle flow cell (Oxford Nanopore technologies) using the Ligation Sequencing Kit SQK-LSK110. The flow cell ran with 75 active sites and produced a total of 216,826 reads. Short and long reads were mapped on the viral contigs using Bowtie2 [34] and Minimap2 [35], respectively, and coverages were determined with SAMtools [36].

The viral termini were first amplified by RT-PCR on CTAB-extracted nucleic acids and then obtained by Sanger sequencing. For the 5’ terminus, the SMARTer RACE 5’/3’ Kit (Takara) was followed in combination with the primer Seq5Two (Table 1). For the 3’ terminus, a poly(U) tailing was first performed using the Poly(U) Polymerase (NEB). The viral 3’ end was then amplified by RT-PCR as previously described [26] with the primers Tymo5closer and AnchoredPolyA (Table 1). For both termini, amplicons were cloned and sequenced as mentioned above.

### Bioinformatics analyses

Viral sequences were analyzed on Ugene [37] and Jalview [38]. Conserved protein domains were identified with the CDD database [39]. RNA secondary structures were predicted as previously described [40]. Proteins and nucleotides were aligned with Muscle [41]. Pairwise identities matrixes for these alignments were generated with the SDT software [42]. Amino acid (AA) contents were obtained with EMBOSS pepstats (available at: https://www.ebi.ac.uk/Tools/seqstats/emboss_pepstats/). AA logos were produced on Seq2logo [43]. For phylogenetic analyses, the best fit substitution models for the protein alignments were first retrieved with ModelFinder [44] and used to create maximum likelihood (ML) phylogenetic trees on IQ-Tree [45] in combination with ultrafast bootstraps [46]. ML trees were curated on ITol [47].

### Transmission assays

For the insect transmission assays, specimens were placed for 24 h in homemade plastic boxes containing detached symptomatic leaves of *M. sylvestris*. Then, pairs of insects were placed onto 3-week old *M. sylvestris* plants covered in insect-proof nets (Andermatt), and carefully removed after one week. For transmission assays using aphids, plants were also treated with the commercial insecticide Tapeki. Plants were then maintained one month in greenhouse for symptom observation. For each plant, the infection status was then determined by RT-PCR.

From one infected plant in Lausanne, seeds were collected in the 2021 autumn and kept at 4 °C until the following spring. Seeds were then sown and the resulting plants were grown in greenhouse conditions for one month. The infection status was then assessed by RT-PCR on pools combining the RNA samples from four plants.

## Results

### A mixed viral infection on hollyhock

**Fig. 1:**
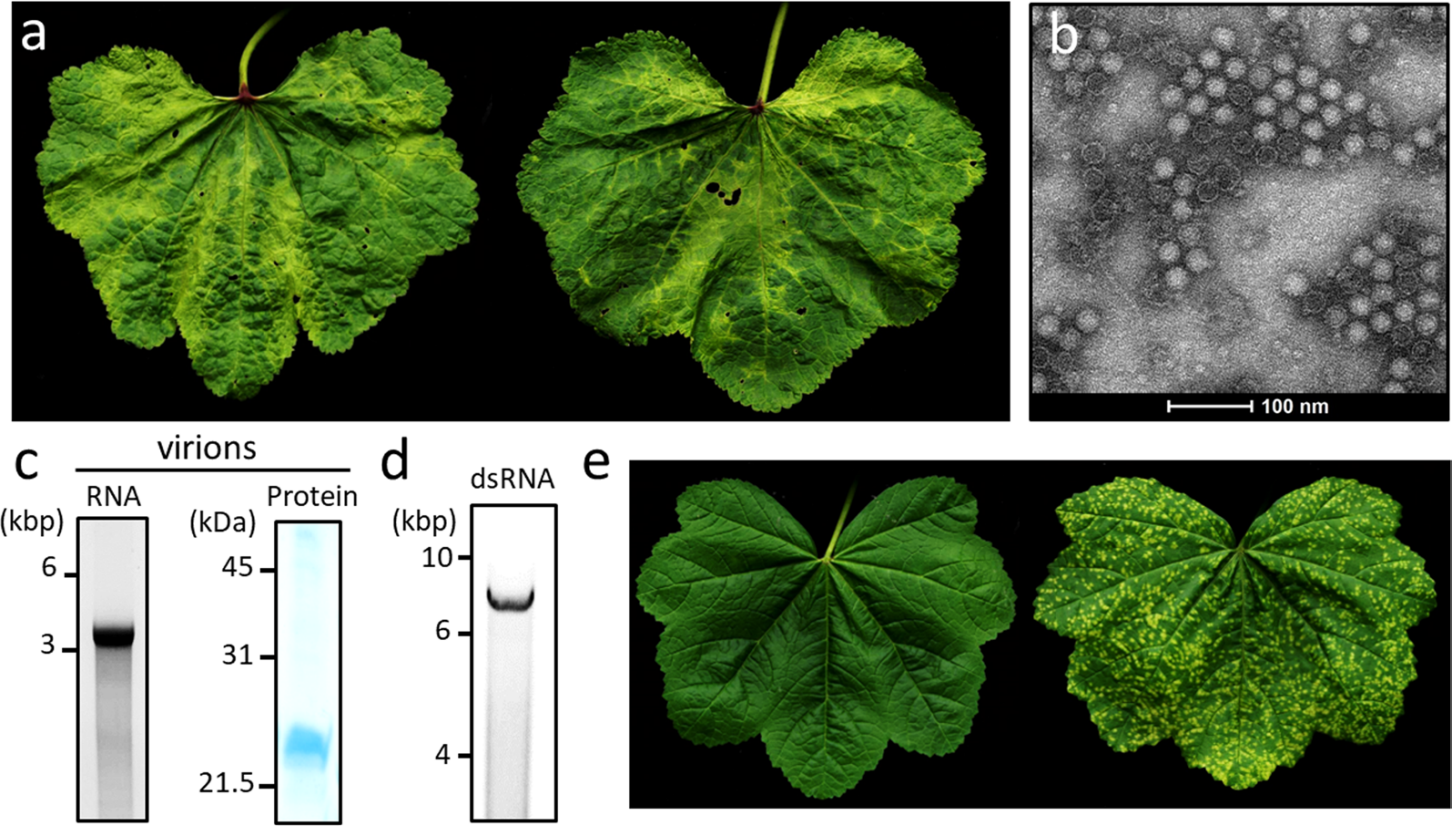
Identification of the novel tymovirus AYMV. **a** Hollyhock leaves collected in Lausanne showing vein clearing, chlorosis and mosaic symptoms. **b** Transmission electron micrograph of semi-purified virions negatively stained with 4% phosphotungstate. Note the staining penetration in empty particles. **c** Electrophoresis gel for RNA (left) and protein (right) associated with semi-purified particles. **d** Electrophoresis gel for the dsRNA extracted from symptomatic hollyhock leaves. **e** Healthy leaf of *M. sylvestris* (left) in comparison with an AYMV-infected leaf showing chlorotic spots (right), at 30 dpi

Symptoms consisting of vein chlorosis and yellow mosaic were noted in May 2021 on leaves of hollyhocks at two public sites in Lausanne and Nyon (Fig. 1a). Leaf samples from both locations were first checked for the presence of putative viral agents by TEM analysis of leaf dip preparations. Numerous isometric particles were visible in samples from both sites, while samples from Nyon contained additional flexuous particles > 800 nm in length (Additional file 2: Fig. S1). These flexuous virions were reminiscent of members of *Potyvirus*, the largest genus of plant + ssRNA viruses, and this was confirmed by RT-PCR analyses using the generic primer pair CIFor/Rev (Table 1). Indeed, a cDNA of *ca.* 400 bp was amplified from RNA samples from Nyon but not from Lausanne. Blastn search on the sequenced amplicon revealed weak homology with the potyvirus Narcissus yellow stripe virus (GenBank acc. number LC314392.1, E-value = 6e−12, 80% nt identity), advocating that a novel potyviral species was present.

In order to gain insights into the isometric particles, a semi-purification protocol was performed on leaves collected in Lausanne. Two types of particles (*ca.* 30 nm in diameter) were clearly identified by TEM, corresponding presumably to intact virions and “empty” shells (Fig. 1b). Electrophoretic analysis of nucleic acid extracted from these particles disclosed a single RNA that migrated slightly above a 3 kbp DNA marker (Fig. 1c, left). A protein of *ca.* 21 kDa was detected following particles denaturation and SDS-PAGE analysis (Fig. 1c, right). In parallel, a dsRNA 6–7 kbp in length was extracted from symptomatic leaves from the same location (Fig. 1d). Altogether, these results were advocating for the presence of a tymovirus [1]. This was tested by RT-PCR on RNA samples from both sites using the generic primer pair TymZ-F/R (Table 1). Both samples yielded a similar 340-bp amplicon that was highly similar to tymoviruses as revealed by Blastn search. The best hit (E-value = 3e−66, 80% nt identity) corresponded to the partially described Watercress white vein virus (WWVV, Genbank acc. number JQ001816.1, [48]), suggesting that a new tymovirus was present.

To investigate the pathogenicity of the novel tymovirus, leaf samples from both sites were used for mechanical inoculations of several indicator plants. Both inoculation procedures led to similar results. Plants of *Nicotiana occidentalis* showed stem curling and vein yellowing at 10 dpi (Additional file 2: Fig. S2a–c). Plants of *N. benthamiana* turned yellow and exhibited mosaic symptoms at 12 dpi (Additional file 2: Fig. S2d). Inoculated leaves of *Chenopodium quinoa* and *C. henopodium amaranticolor* showed local lesions after one week (Additional file 2: Fig. S2e), but no systemic symptom was recorded on these species. Neither local nor systemic symptoms were visible on *N. clevelandii*, *N. tabacum* and *Brassica rapa* ssp. *chinensis*. Intriguingly, only RNA samples from *C. quinoa* and *C. amaranticolor* tested positive for the potyvirus by RT-PCR when inoculated with samples from Nyon and no sample tested positive for the tymovirus (Additional file 2: Fig. S3). The symptoms observed on *N. occidentalis* and *N. benthamiana* in which the potyvirus and the tymovirus were not detected were thus likely associated with another virus. TEM analysis of leaf dip preparations from these plants revealed flexuous particles *ca.* 600 nm in length (Additional file 2: Fig. S4). RNA samples from these plants were thus tested by RT-PCR using the primer pair CarF2b/R targeting members of *Carlavirus* (Table 1), a large genus of flexuous + ssRNA viruses. The RT-PCR yielded an amplicon of *ca.* 750 bp, corresponding to a fragment of Gaillardia latent virus (GLV, Genbank acc. number LC485538.1, 96% nt identity, [49]). GLV was detected in all symptomatic indicator plants as well as the collected hollyhocks from both sites (Additional file 2: Fig. S3).

Some tymoviruses have narrow host ranges limited to the family of their natural host [50, 51], and this trait could explain the lack of transmission of the newly identified tymovirus on indicator species. Particles purified by ultracentrifugation from leaves collected in Lausanne were thus mechanically inoculated on the common mallow (*Malva sylvestris*), a fast-growing relative of hollyhock. No local symptom was observed but chlorotic spots and yellowing appeared at 12 dpi on upper leaves (Fig. 1e). RT-PCR analyses showed the presence of the tymovirus and the absence of the potyvirus and GLV (Additional file 2: Fig. S5). The tymovirus was thus hereafter named “Alcea yellow mosaic virus” (AYMV). Inoculations of *M. sylvestris* plants with GLV only (isolated on *N. occidentalis*) or GLV in combination with the potyvirus (isolated on *C. quinoa*) lead to symptomless infection and yellowing, respectively (data not shown).

### Genomic characterization of AYMV

Given the symptoms observed on the common mallow, AYMV-infected leaves of this plant were used for an extraction of viral dsRNA followed by HTS. Following Illumina sequencing and de novo assembly, numerous contigs showing significant similarities to tymoviruses were yielded (mean coverage = 357x, Additional file 2: Fig. S6a) while none of them indicated the presence of any other virus. These contigs were then bridged by RT-PCR and Sanger sequencing. However, one region amplified with the primer pair TymoFinalF/R (Table 1, positions 1826–2644) could neither be sequenced by Sanger sequencing nor cloned into the pGEM-T plasmid despites numerous attempts (data not shown). This amplicon was eventually sequenced by long read sequencing on a Nanopore Flongle flowcell (mean coverage = 80x, Additional file 2: Fig. S6b). Following Sanger sequencing of the termini, a full-length genome was eventually compiled for AYMV and submitted to Genbank under accession number OP227146.

**Fig. 2:**
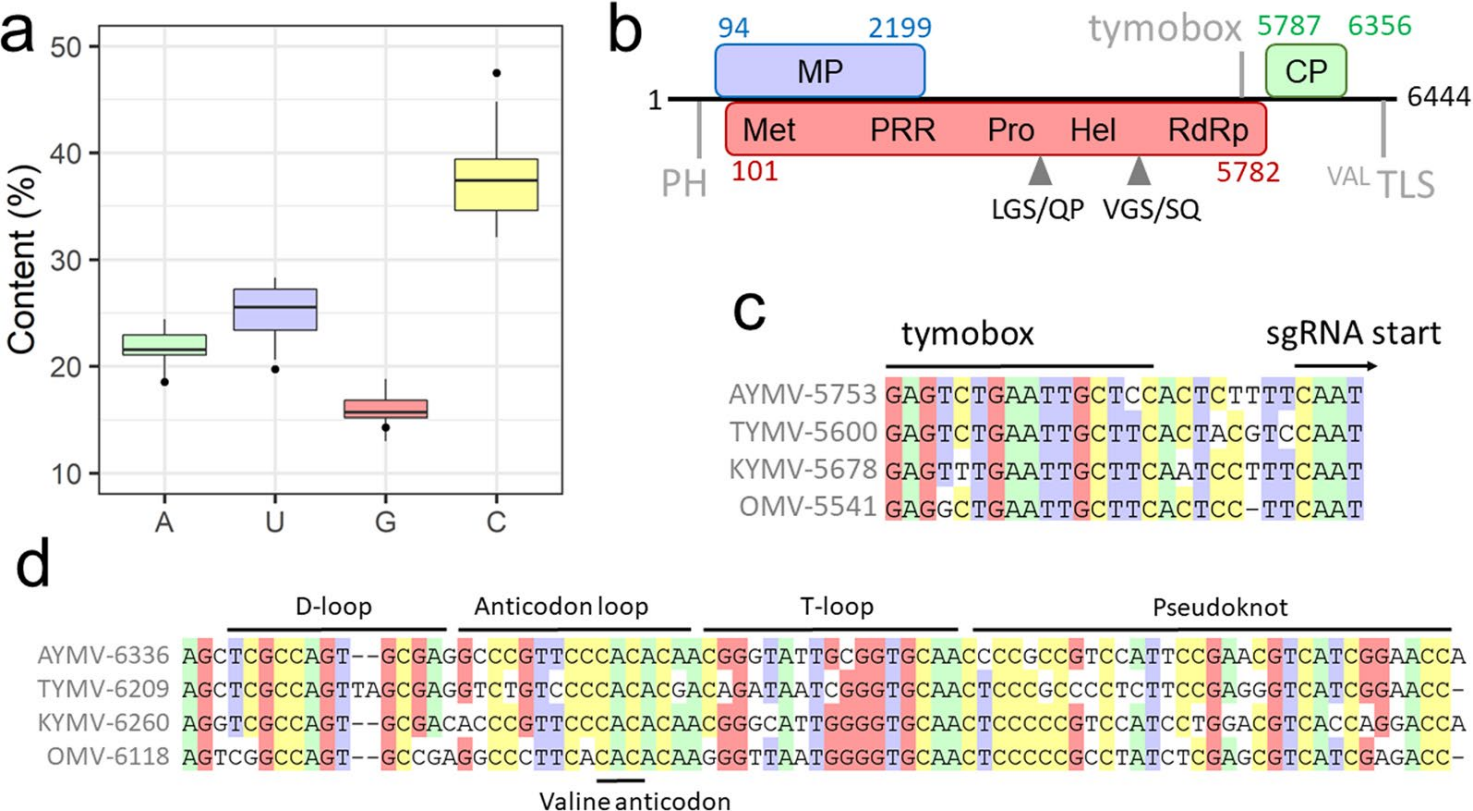
Characterization of AYMV genome. **a** Base content for the gRNA of tymoviruses (box plots) and AYMV (black points). **b** Schematic representation of AYMV gRNA. ORFs are represented as colored boxes with associated positions and conserved domains/functions (see main text for details). The putative RP cleavage sites are indicated by gray arrows. The protonable hairpin (PH), tymobox and valine tRNA-like structure (^Val^TLS) are indicated by vertical gray lines. **c** Alignment of the tymobox cDNA for AYMV and selected related species. **d** Alignment of the 3’-UTR cDNA for the same viruses described in Fig. 2c. The structural annotations are reproduced from a previous analysis of tymoviral tRNA (52). Colored background indicate ≥ 75% conservation. Full virus names and accession numbers are listed in Additional file 1: Table S1

The length of AYMV gRNA is 6444 nt, and its 5′ and 3′ untranslated regions (UTR) are 93 and 88 nt long, respectively (Fig. 2b). Tymoviral genomes were previously shown to be highly rich in cytidine [17], and this trait is exceptionally marked in the case of AYMV gRNA with 47.5% of cytidine (Fig. 2a). In particular, the region that resisted Sanger sequencing and bacterial cloning is made of 58% of cytidine with long repetitive stretches.

AYMV gRNA exhibits other features typical of tymoviruses: a protonable hairpin with internal C-A mismatches was identified at position 12–40 within the 5’-UTR (Fig. S7, ΔG = − 6.20 kcal/mol) and the tymobox was found starting at position 5,753, harboring one mismatch with the TYMV tymobox (Fig. 2c). AYMV 3’-UTR is likely to fold into a valine-accepting TLS given the extensive similarities with structural domains found in the 3’-UTRs of related viruses (Fig. 2d). The gRNA ends with a CCA motif which is presumably involved in replication [17]. Consistent with other tymoviruses, three ORFs encoding for MP, RP and CP were identified on AYMV genome (Fig. 2b, colored boxes).

AYMV MP is 702 AAs in length and shows low percentages of pairwise identities with other tymoviral MPs (30–43%, Fig. S8). A search in the CDD database failed to detect any conserved domain for this protein. It is particularly rich in proline (P, 25.9%) and leucine (L, 13.9%), and to a lesser extent in serine (S, 11.3%) and arginine (R, 9.0%) residues, and this AA bias is also observed for other tymoviral MPs (Additional file 2:Fig. S9). In these proteins, the N-terminal moiety is more conserved than the C-terminal moiety, and this region harbors several conserved motifs and positively-charged AAs (Additional file 2: Figs. S10–S11). Unlike tymoviral CPs and RPs, MPs do not have direct homolog in *Marafivirus* and *Maculavirus*, the two other *Tymoviridae* genera. A few viruses of these genera harbor ORFs overlapping with RP, but no translation product has been evidenced and their role has not been established [53]. In addition, these putative proteins do not contain the conserved motifs detected in tymoviral MPs and are much shorter (data not shown). Potential remote homologs of tymoviral MPs were searched using HHBlits [54], but only the aforementioned proteins of maculaviruses and marafiviruses were yielded, in line with the idea that tymoviral MPs are unique in the known global virome [55].

AYMV RP is 1894 AAs in length and shares 52–65% AA identities with other tymoviral RPs (Fig. S12, left). The following domains were predicted on this protein: Met pfam01660 (positions 39–349, E-value = 1.18e−74), Pro cl05113 (positions 826–919, E-value = 1.21e−18), Hel pfam01443 (positions 1018–1255, E-value = 2.50e−53) and RdRp pfam00978 (position 1480–1827, E-value = 0). In addition, the domains Met and Pro are separated by a proline-rich region (PRR, with 24.4% of P) which corresponds to the region recalcitrant to Sanger sequencing and cloning. Based on previous studies [20], putative cleavage sites in the RP were identified at the motifs ^924^LGS/QP and ^1307^VGS/SQ (Fig. 2b). AYMV CP is 189 AAs in length, shares 33–64% identities with other tymoviral CPs (Additional file 2: Fig. S12, left) and harbors the tymovirus coat domain pfam00983 (E-value = 1.10e−35) at positions 21–183.

### Phylogenetic analyses

**Fig. 3:**
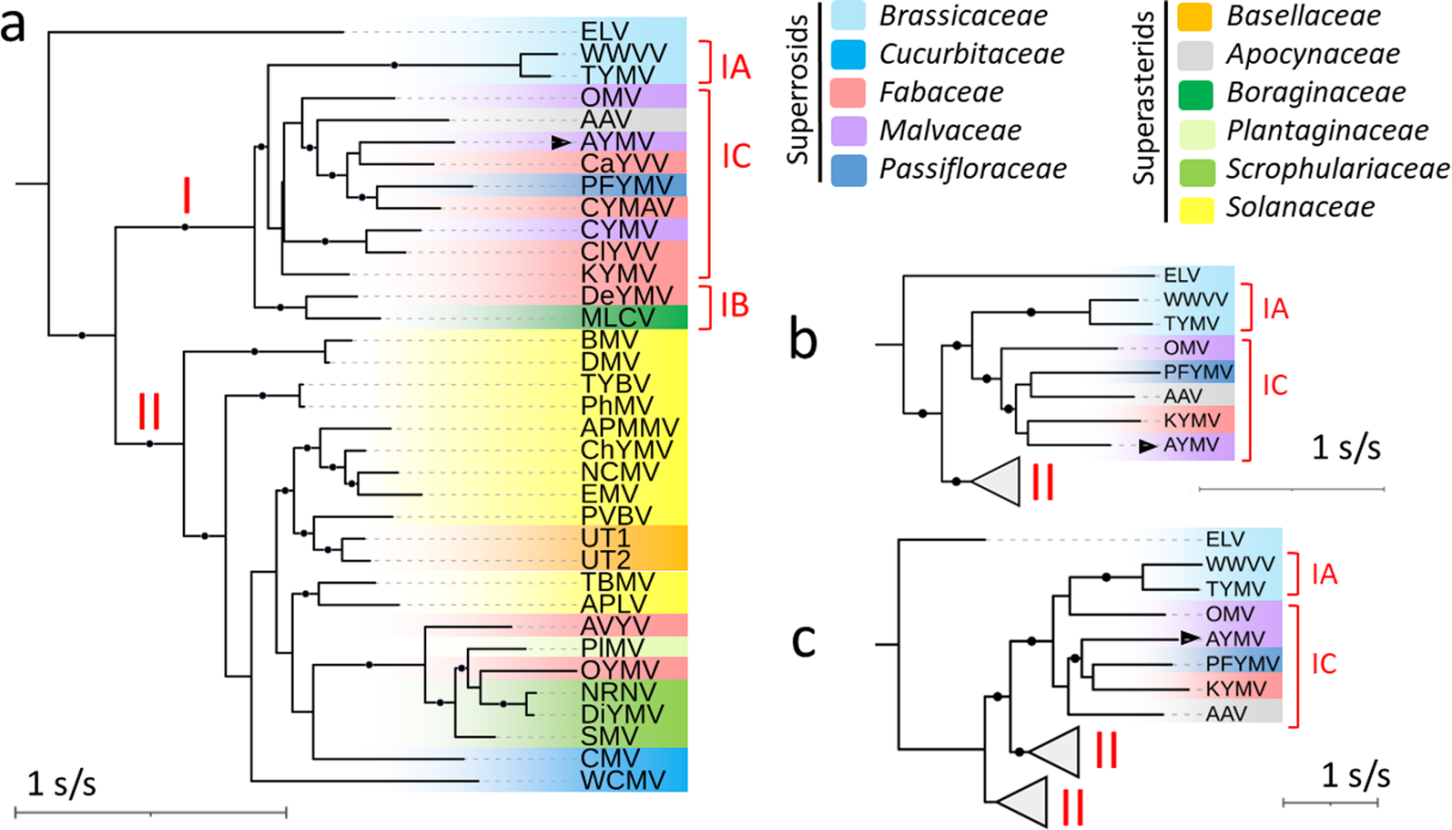
ML phylogenetic tree of the tymoviral proteins. **a** CP tree (model: rtREV + F + I + G4). **b** RP tree (model: rtREV + F + I + G4). **c** MP tree (model cpREV + F + I + G4). Full virus names and accession numbers are listed in Additional file 1: Table S1. The position of AYMV is highlighted by a black arrow. Selected members of the genera *Marafivirus* and *Maculavirus* were used to root the initial CP and RP trees. Black circles on branches indicate bootstrap support > 70%. The tree scales are given in substitution per site. Colored background are indicative of the family of the natural host

Among the three tymoviral proteins, the CP allows for the most inclusive phylogenetic analysis because 34 sequences for this protein have been deposited on NCBI (Additional file 1: Table S1, excluding sequences from metagenomics data), while only 25 sequences are available for MPs and RPs. This is due to the use of generic primers amplifying the 3’ region of gRNAs. A first ML phylogenetic tree was therefore constructed for an alignment of tymoviral CPs (Fig. 3, left). The topology of the obtained tree is in line with early serological studies that have evidenced a clear division of *Tymovirus* in two main clades (hereafter termed clade I and II), with the exception of the highly divergent Erysimum latent virus (ELV) [56]. Clade I accommodates TYMV and other members mainly found on superrosids and for which fabaceous plants represent the largest group of natural hosts. Clade II contains viruses mainly retrieved from superrasterids, and in particular solanaceous plants. Members of clade I are grouped in two strongly supported subclades (IA and IB, 100% bootstrap replicates) and a third, less-supported subclade (IC, 68% bootstrap replicates). AYMV clusters with members of the latter which also gathers the other mallow-infecting tymoviruses OMV and CYMV, four viruses found on *Fabaceae* as well as two viruses found on plants from the families *Passifloracea* and *Apocynaceae*.

ML trees were then inferred for alignments of RPs and MPs (Fig. 3b–c). The topology of the RP tree is similar to the CP tree, exhibiting the same robust grouping into clades I and II. In this tree, AYMV is placed again within subclade IC, which is more strongly supported than in the CP tree. The MP tree differs by having members of clade II and subclade IC no longer hold into monophyletic groups. Nevertheless, AYMV is still most closely related to viruses from subclade IC in this tree.

### Biological characterizations of AYMV

An experimental host range was assessed for AYMV based on sap inoculations. The virus was found to systemically infect all the tested mallows but not species from ten other families (Additional file 1: Table S2). On susceptible hosts, mechanical inoculation was highly effective as all plants became symptomatic. Local lesions were rarely observed and symptoms typically appeared on upper leaves, consisting mostly in vein yellowing, mosaic, and chlorotic spots sometimes turning necrotic. The most severe symptoms were recorded on the common mallow and lavatera (*Lavatera trimestris*), on which most systematically infected leaves eventually became totally necrotic and fell off the plants. Notably, systemic infections were noted on the agronomically relevant mallows okra and cotton, on which pale green mottling were observed.

**Fig. 4:**
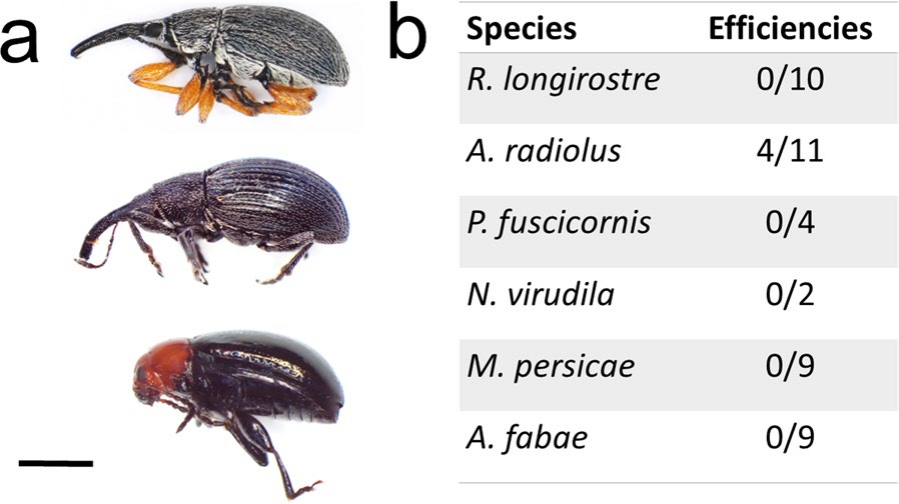
Insect transmission assays. **a** Specimens of *Coleoptera* found on hollyhock and used for transmissions assay. Up: *R. longirostre*, middle: *A. radiolus* and low: *P. fuscicornis*. The bar represents 1 cm. **b** Transmission efficiencies for the different insect species. Pairs of insects were used for each transmission assay

Specimens belonging to the weevil species *Aspidapion radiolus* and *Rhopalapion longirostre,* (*Coleoptera: Curculionoidea*) and the mallow flea beetle *Podagrica fuscicornis* (*Coleoptera: Chrysomelidae*) were collected on healthy hollyhocks in a private garden in Prangins (district of Nyon, Switzerland) as they represent chewing insect that could serve as vector (Fig. 4a). In addition, specimens of the stink bug *Nezara viridula* (*Hemiptera: Pentatomidae*) were also collected from the same plants. These insects were used for transmission experiments, together with specimens of the aphid species *Myzus persicae* and *Aphis fabae* (*Hemiptera*: *Aphididae*). Even though all insects fed on plants, as evidenced by visible damages, transmission of AYMV was only observed with *A. radiolus* for which four plants became symptomatic and tested positive by RT-PCR among the 11 tested (Fig. 4b). In parallel, vertical transmissions was not recorded for 32 tested hollyhocks, suggesting that AYMV is not seed-borne.

## Discussion

Here, diagnosis tools were used to untangle mixed infections in symptomatic hollyhocks collected at green sites from two Swiss cities, revealing the presence of three distinct + ssRNA viruses. Early results showed that AYMV was present at both sites and indicated that this virus represented the first isolate of a new species, which prompted us to study this virus in particular.

We first aimed to obtain a full-length sequence for AYMV genome based on short-read sequencing of purified viral dsRNA together with bridging RT-PCR analyses. However, long-read sequencing was required to close the genome as a region could not be covered by short-read assembly and was recalcitrant to Sanger sequencing as well as bacterial cloning. The latter issue has been already described during the cloning of a similar region for the other tymovirus eggplant mosaic virus [57]. This might have been caused by the high content in cytidine in the recalcitrant region, present as long repetitive stretches known to be associated with sequencing issues [58]. The overall level of cytidine is exceptionally high in AYMV gRNA, representing the record among tymoviruses. The reason for this strong nucleotide bias is unknown but could be related to efficient encapsidation. Indeed, under the light-induced, low-pH conditions found in the chloroplast-derived replication vesicles of TYMV, stretches of poly-cytidine have been hypothesized to form tertiary structures required for virion assembly [21]. The high cytidine content is also directly involved in the production of numerous proline residues (encoded by CCC codons) in the MP and RP. It was recently shown that TYMV MP acts as a VSR by inhibiting the amplification step of the antiviral silencing, and co-localizes with siRNA bodies [59]. One can speculate that the proline residues in tymoviral MPs are involved, together with the conserved motifs and positively-charged AAs, in specific protein–protein or protein-RNA interactions within those bodies.

We identified other molecular and genomic features for AYMV that clearly associate this virus with tymoviruses, and phylogenetic trees further confirmed a robust positioning inside the genus. As illustrated here by the grouping of AYMV and other mallow-infecting members within subclade IC (Fig. 3), previous studies have highlighted the association between serological groups, natural hosts and host ranges among tymoviruses [56]. However, despites several attempts, the molecular bases underlying this trait have not been found yet [57, 60].

The identification of a weevil vector for AYMV is rather interesting because, so far, Scrophularia mottle virus was the only member of the genus known to be transmitted by insects of this group [61]. Most described vectors of tymoviruses are flea beetles from the genera *Epitrix* and *Diabrotica*, but other leaf-eating insects have been evidenced as vectors as well [24, 62, 63]. Transmission assays have highlighted short acquisition time and long retention time, leading to the consensus that tymoviruses are likely “semi-persistently” transmitted in a mechanical process [13, 24]. However, such a transmission mode still implies vector specificity, as noted here by the lack of transmission of AYMV based on the results using 20 individuals of *R. longirostre*. In comparison to other insect groups, little is known regarding the spread of beetle vectors of plant viruses [64]. It can be hypothesized that *A. radiolus* had been involved in the dissemination of AYMV in the two collection sites. Alternatively, given the high transmissibility of the virus, it is possible that it was carried on contaminated material via another unknown vector or through human activities.

In summary, in this study we have described the novel virus AYMV found in mixed infection on hollyhock in Switzerland. In the genus *Tymovirus*, the threshold for species demarcation established by the International Committee on Taxonomy of Viruses is fixed at 90% AA identity for the CP and 80% nt identity for the genomic RNA [65]. The closest homolog to AYMV CP is CaYVV CP (64.5% AA identity) and the closest genome is TYMV-HS16 (KP995418.1, 14% coverage, 78.6% nt identity), advocating altogether that AYMV should be considered as the first isolate of a new viral species. Additional molecular and genomic analyses on GLV and the novel potyvirus will help better characterize these viruses as well as their interaction with AYMV. Since hollyhocks are widely distributed, these plants may serve as important reservoir for crop viruses, as noted by other studies [66, 67]. In the case of AYMV, symptomatic infections were evidenced on okra and cotton, advocating that this virus might represent a threat for these important crops should it be introduced in their respective cultivation areas.

## Supplementary Information

**Additional file 1:** Supplementary figures.

**Additional file 2:** Supplementary tables.

## Data Availability

Not applicable.

## Abbreviations

AA: Amino acid
AYMV: Alcea yellow mosaic virus
CYMV: Cacao yellow mosaic virus
CP: Capsid protein
dsRNA: Double-stranded RNA
dpi: Days post inoculation
ELV: Erysimum latent virus
gRNA: Genomic RNA
gRNA: Genomic RNA
GLV: Gaillardia latent virus
Hel: Helicase
HTS: High-throughput sequencing
ML: Maximum likelihood
Met: Methyltransferase
MP: Movement protein
OMV: Okra mosaic virus
Pro: Papain-like protease
+ ssRNA: Positive-sense, single-stranded RNA
RP: Replication protein
RdRp: RNA-dependent RNA polymerase
sgRNA: Subgenomic RNA
TBMV: Tomato blistering mosaic virus
TEM: Transmission electron microscopy
tRNA: Transfer RNA
TLS: tRNA-like structure
TYMV: Turnip yellow mosaic virus
UTR: Untranslated regions
VSR: Viral suppressor of RNA silencing
