# Supplementary material for: A novel weevil-transmitted tymovirus found in mixed infection on hollyhock
Source: Virol J. 2023 Jan 30;20:17. doi: 10.1186/s12985-023-01976-6 (PMC9885571; doi:10.1186/s12985-023-01976-6)
Supplement: Supplementary file 1 — Additional file 1: Supplementary figures. [file 12985_2023_1976_MOESM1_ESM.pdf]

**Table S1:** Accession numbers for the viral genomes and proteins used in this study.

| Virus name | Accr. | Genome | MP | RdRp | CP |
|---|---|---|---|---|---|
| ***Tymovirus*** | | | | | |
| *Anagyris vein yellowing virus* | AVYV | NC_011559.1 | YP_002308577.1 | YP_002308578.1 | YP_002308579.1 |
| *Andean potato latent virus* | APLV | NC_020470.1 | YP_007517179.1 | YP_007517180.1 | YP_007517181.1 |
| *Andean potato mild mosaic virus* | APMMV | NC_020471.1 | YP_007517182.1 | YP_007517183.1 | YP_007517184.1 |
| *Asclepias asymptomatic virus* | AAV | NC_015523.1 | YP_004464923.1 | YP_004464924.1 | YP_004464925.1 |
| *Belladonna mottle virus* | BMV | | | | YP_009508097.1 |
| *Cacao yellow mosaic virus* | CYMV | | | | YP_009508099.1 |
| *Calopogonium yellow vein virus* | CaYVV | | | | AAC58458.1 |
| *Cassia yellow mosaic-associated virus* | CYMAV | | | | AEX10524.1 |
| *Chayote mosaic virus* | CMV | NC_002588.1 | NP_067736.1 | NP_067737.1 | NP_067738.1 |
| *Chiltepin yellow mosaic virus* | ChYMV | NC_014127.1 | YP_003620400.1 | YP_003620401.1 | YP_003620402.1 |
| *Clitoria yellow vein virus* | ClYVV | | | | YP_009505640.1 |
| *Desmodium yellow mottle virus* | DeYMV | | | | YP_009505641.1 |
| *Diascia yellow mottle virus* | DiYMV | NC_011086.1 | YP_002048672.1 | YP_002048673.1 | YP_002048674.1 |
| *Dulcamara mottle virus* | DMV | NC_007609.1 | YP_406374.1 | YP_406375.1 | YP_406376.1 |
| *Eggplant mosaic virus* | EMV | NC_001480.1 | NP_040967.1 | NP_040968.1 | NP_040969.1 |
| *Erysimum latent virus* | ELV | NC_001977.1 | NP_047919.1 | NP_047920.1 | NP_047921.1 |
| *Kennedya yellow mosaic virus* | KYMV | NC_001746.1 | NP_044327.1 | NP_044328.1 | NP_044329.1 |
| *Mertensia leaf curl virus* | MLCV | | | | ACN62330.1 |
| *Naranjilla chlorotic mosaic virus* | NCMV | MG323924.1 | AUR53411.1 | AUR53413.1 | AUR53412.1 |
| *Nemesia ring necrosis virus* | NRNV | NC_011538.1 | YP_002308441.1 | YP_002308442.1 | YP_002308443.1 |
| *Okra mosaic virus* | OMV | NC_009532.1 | YP_001285471.1 | YP_001285472.1 | YP_001285473.1 |
| *Ononis yellow mosaic virus* | OYMV | NC_001513.1 | NP_041256.1 | NP_041257.1 | NP_041258.1 |
| *Passion fruit yellow mosaic virus* | PFYMV | KY823429.1 | AVW89219.1 | AVW89220.1 | AVW89221.1 |
| *Petunia vein banding virus* | PVBV | | | | YP_009664769.1 |
| *Physalis mottle virus* | PhMV | NC_003634.1 | NP_619755.1 | NP_619756.1 | NP_619757.1 |
| *Plantago mottle virus* | PlMV | NC_011539.1 | YP_002308444.1 | YP_002308445.1 | YP_002308446.1 |
| *Scrophularia mottle virus* | SMV | NC_011537.1 | YP_002308438.1 | YP_002308439.1 | YP_002308440.1 |
| *Tomato blistering mosaic virus* | TBMV | NC_021851.1 | YP_008318041.1 | YP_008318042.1 | YP_008318043.1 |
| *Tomato yellow blotch virus* | TYBV | EU779803.2 | AEP40394.1 | AEP40395.1 | ACI96295.1 |
| *Turnip yellow mosaic virus* | TYMV | NC_004063.1 | NP_663296.1 | NP_663297.1 | NP_663298.1 |
| *Ullucus tymovirus 1* | UTV1 | MH645153.1 | AZF99026.1 | AZF99027.1 | AZF99028.1 |
| *Ullucus tymovirus 2* | UTV2 | MH645152.1 | AZF99023.1 | AZF99024.1 | AZF99025.1 |
| *Watercress white vein virus* | WWVV | JQ001816.1 | AFC95825.1 | AFC95826.1 | AFC95827.1 |
| *Wild cucumber mosaic virus* | WCMV | | | | YP_009664771.1 |
| ***Marafivirus*** | | | | | |
| *Grapevine asteroid mosaic associated virus* | GAMAV | NC_031692.1 | | YP_009315883.1 | YP_009315885.1 |
| *Oat blue dwarf virus* | OBDV | NC_001793.1 | | NP_044447.1 | NP_044448.1 |
| ***Maculavirus*** | | | | | |
| *Grapevine Red Globe virus* | GRGV | MZ344581.1 | | UCJ01162.1 | UCJ01163.1 |
| *Fig fleck-associated virus* | FFAV | NC_015229.1 | | YP_004300278.1 | YP_004300303.1 |

**Table S2:** Host range of AYMV based on sap inoculations.

| Species | Symptoms | | RT-PCR* |
|---|---|---|---|
| | Local | Systemic | |
| ***Solanaceae*** | | | |
| *Nicotiana benthamiana* | ns | ns | - |
| *Nicotiana tabacum* var. Xanthi | ns | ns | - |
| *Nicotiana occidentalis* | ns | ns | - |
| *Nicotiana clevendii* | ns | ns | - |
| *Solanum lycopersicum* | ns | ns | nt |
| *Solanum melongena* | ns | ns | nt |
| | | | |
| ***Chenopodiaceae*** | | | |
| *Chenopodium amaranticolor* | ns | ns | - |
| *Chenopodium quinoa* | ns | ns | - |
| | | | |
| ***Malvaceae*** | | | |
| *Abelmoschus esculentus* (okra) | ns | CL, VY, PGM | + |
| *Alcea rosea* var. Majorette | CS | CL | + |
| *Gossypium* sp. (cotton) | ns | CL, PGM | + |
| *Hibiscus trionum* var. Naralis | Ns | CL | + |
| *Lavatera trimestris* | CS | CL, N | + |
| *Malva moschata* | CS | CL, N | + |
| *Malva sylvestris* | ns | CL, VY, N | + |
| | | | |
| ***Brassicacea*** | | | |
| *Brassica rapa ssp. chinensis* | ns | ns | - |
| *Sinapis alba* | ns | ns | nt |
| | | | |
| ***Amaranthaceae*** | | | |
| *Atriplex hortensis* | ns | ns | nt |
| *Beta vulgaris* | ns | ns | nt |
| *Beta macrocarpa* | ns | ns | nt |
| | | | |
| ***Cucurbitaceae*** | | | |
| *Cucumis sativus* | ns | ns | nt |
| | | | |
| ***Euphorbiaceae*** | | | |
| *Mercurialis annua* | ns | ns | nt |
| | | | |
| ***Lamiaceae*** | | | |
| *Agastache mexicana* | ns | ns | nt |
| | | | |
| ***Fabaceae*** | | | |
| *Vicia faba* | ns | ns | nt |
| *Phaseolus vulgaris* | ns | ns | nt |
| | | | |
| ***Asteraceae*** | | | |
| *Lactuca sativa* | ns | ns | nt |
| | | | |
| ***Poaceae*** | | | |
| *Hordeum vulgare* | ns | ns | nt |
| *Zea mays* | ns | ns | nt |

ns : no symptom; NS: necrotic spots; CL: chlorotic lesions; CS: chlorotic spots; VY: vein yellowing; PGM: pale green mottling, N: necrosis. *RT-PCR analysis in upper, non-inoculated leaves. “nt”: not tested.
