# Supplementary material for: A novel weevil-transmitted tymovirus found in mixed infection on hollyhock
Source: Virol J. 2023 Jan 30;20:17. doi: 10.1186/s12985-023-01976-6 (PMC9885571; doi:10.1186/s12985-023-01976-6)
Supplement: Supplementary file 2 — Additional file 2: Supplementary tables. [file 12985_2023_1976_MOESM2_ESM.pdf]

Supplementary figures

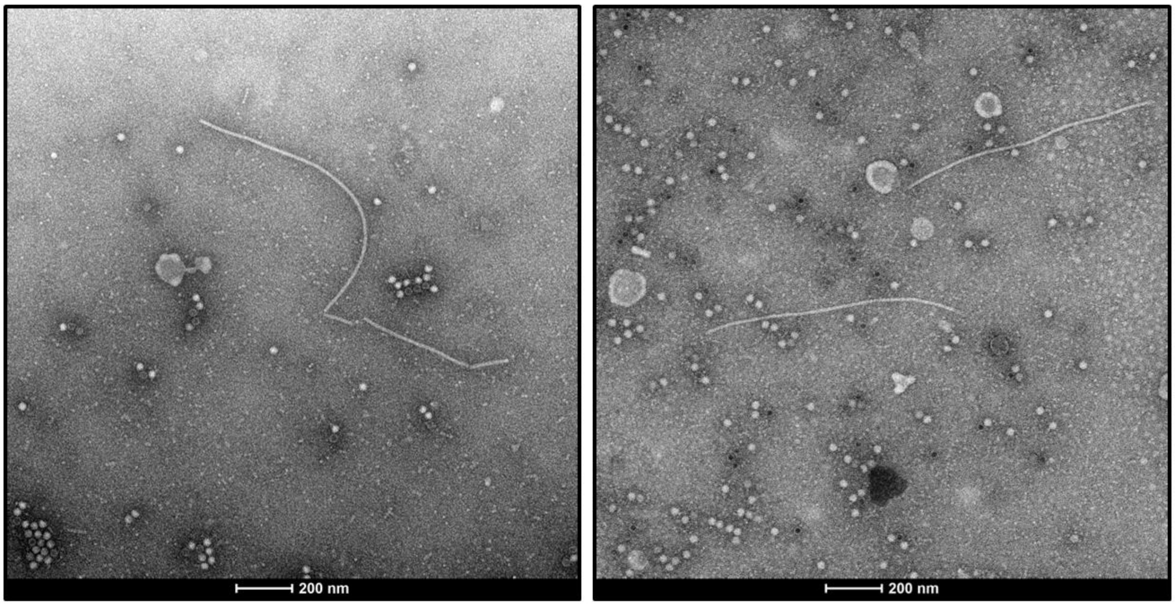

Figure S1. Electron micrographs showing the mix of flexuous and isometric particles detected in leaf samples collected in Nyon.

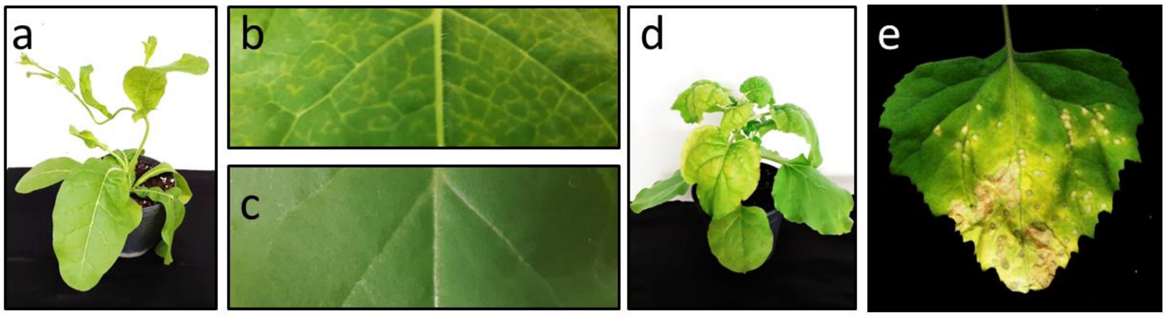

Figure S2: Symptomatic plants for the preliminary sap inoculation assay. Plant of *N. occidentalis* showing stem curling (a) and vein yellowing (b) in comparison to a healthy leaf (c) . Plant of *N. benthamiana* showing yellowing (d). Local chlorotic spots on *C. amaranicolor* (e).

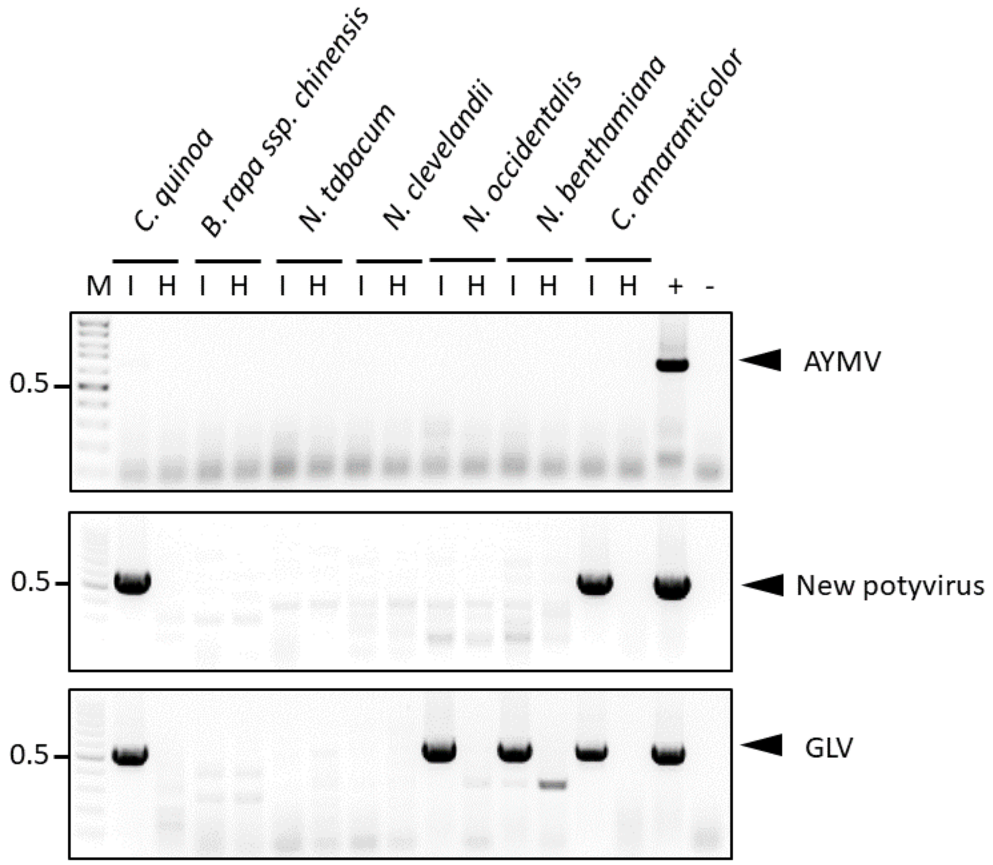

Figure S3: RT-PCR detection of AYMV, GLV and the novel potyvirus in RNA samples from the plants of the sap inoculation assay. The position of the 0.5 kbp is indicated on each gels. For each plant species, "I" refers to inoculated plant while "H" refers to healthy control. Samples were taken from the inoculated leaf in the case of *C. quinoa*, *C. amaranticolor*, while samples were taken from upper leaves for the other species. The positive control "+" corresponds to a sample from hollyhock collected in Nyon. M: 100bp DNA ladder.

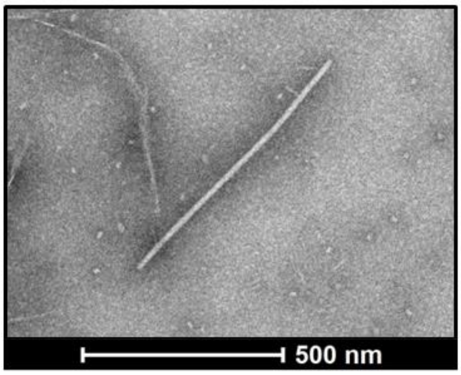

Figure S4: Electron micrograph of a GLV particle visible in leaf dip assays from infected *N. occidentalis*. Virions were negatively stained with with 4% phosphotungstate.

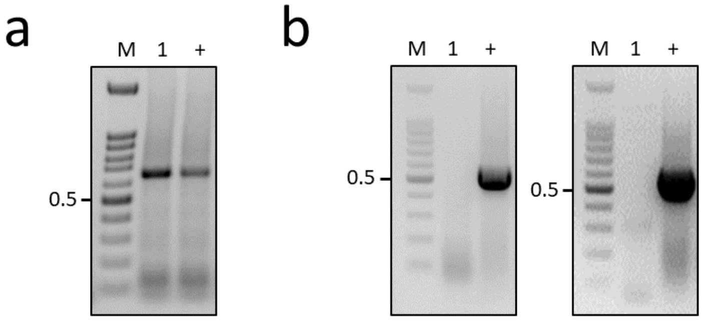

Figure S5: RT-PCR detection of AYMV (a) and GLV (b, left) and the novel potyvirus (b, right) in RNA sample from the *M. sylvestris* plant inoculated with AYMV particles (1) or from positive control samples (+, sample from hollyhock collected in Nyon). The position of the 0.5 kbp is indicated on each gels. M: 100bp DNA ladder.

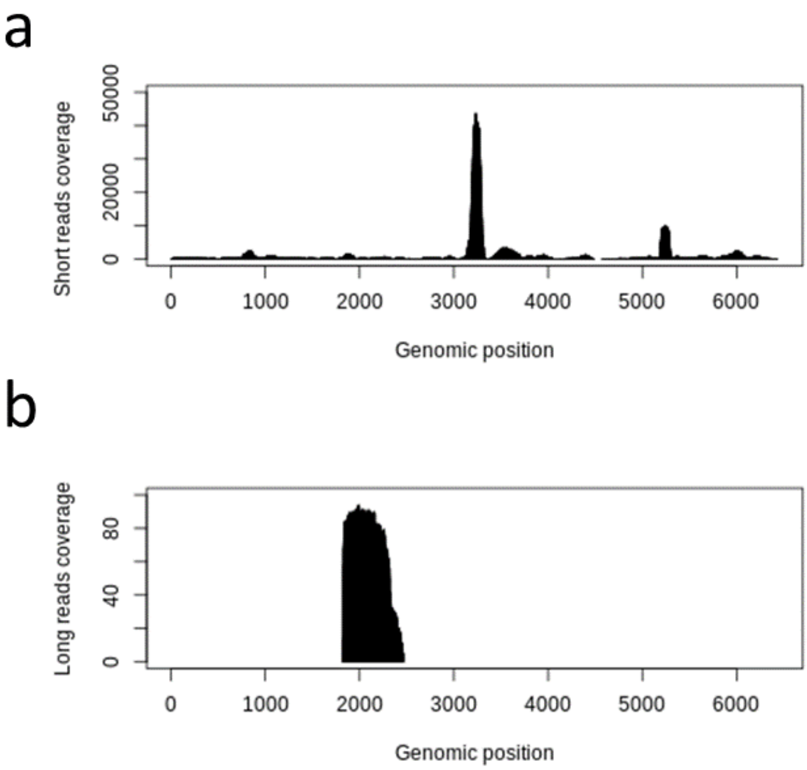

Figure S6: Read coverages obtained after remapping the Illumina reads **(a)** or Nanopore reads **(b)** on AYMV genome.

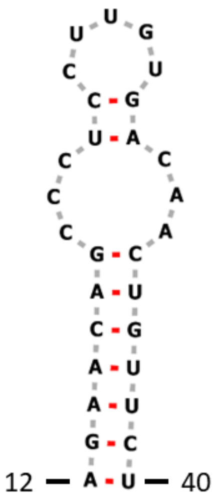

Figure S7. Hairpin structure containing protonable C-A mismatches predicted in the AYMV 5'-UTR.

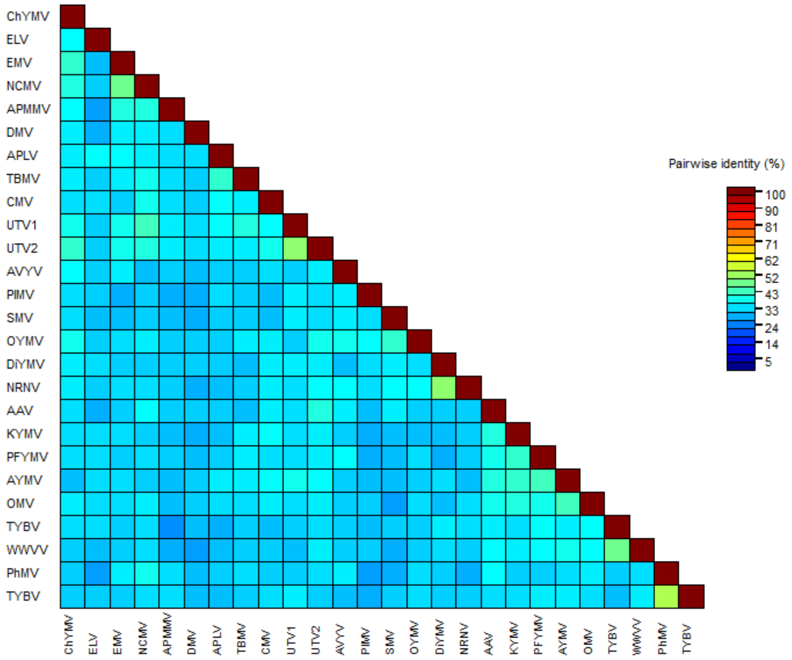

Figure S8. Pairwise identity matrix for tymoviral MPs.

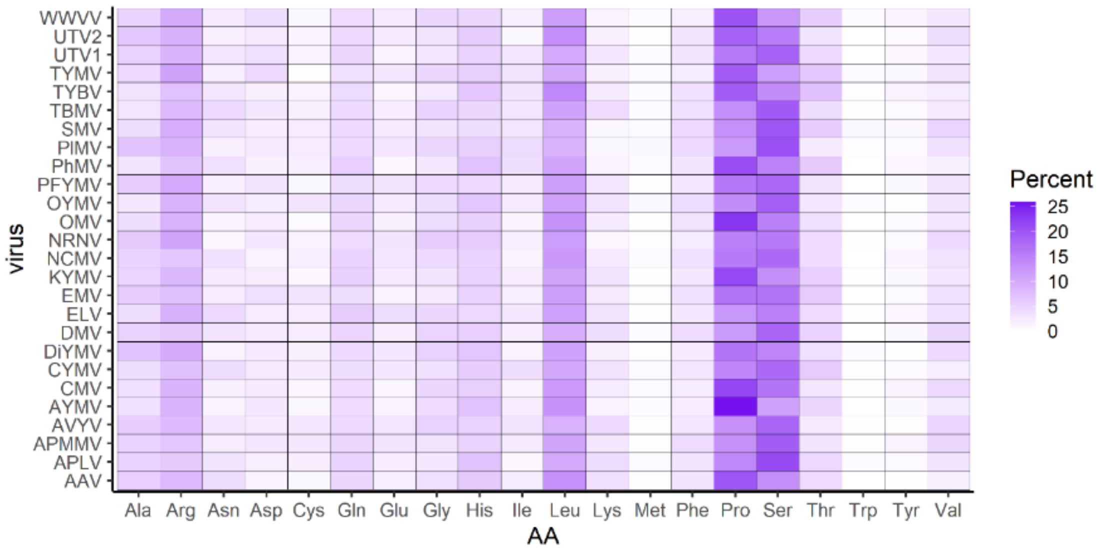

Figure S9. AA content (in percent of total AA) for tymoviral MPs.

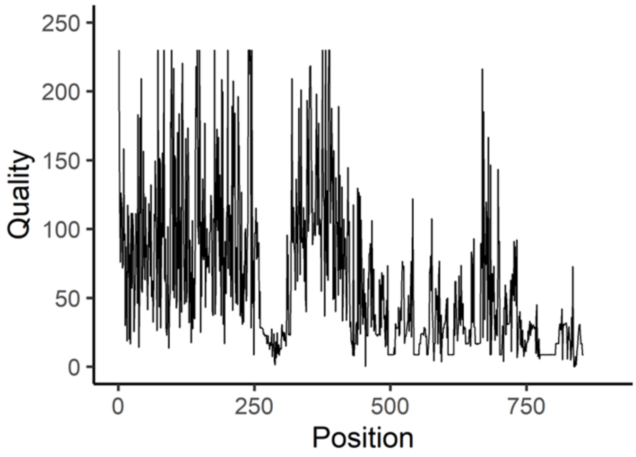

Figure S10. Alignment quality based on Blosum62 score matrix for an alignment of tymoviral MPs. Scores were retrieved from Jalview.

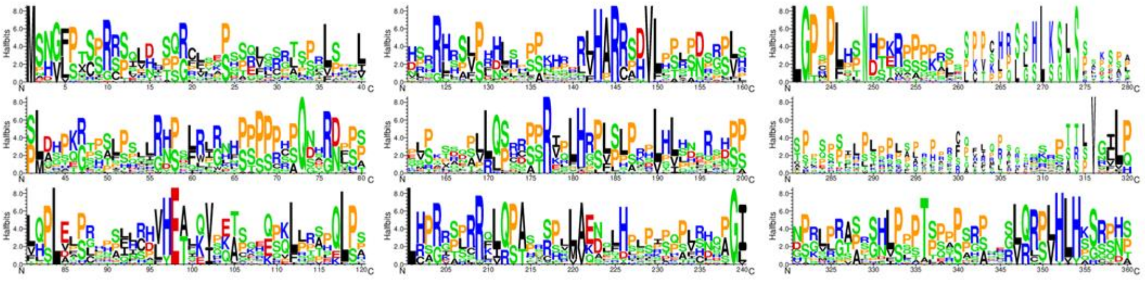

Figure S11. AA sequence logo for the first 360 positions of an alignment of tymoviral MPs. Gold: proline (P); blue: positively-charged AAs; red: negatively-charged AAs; green: polar AAs; black: non polar AAs.

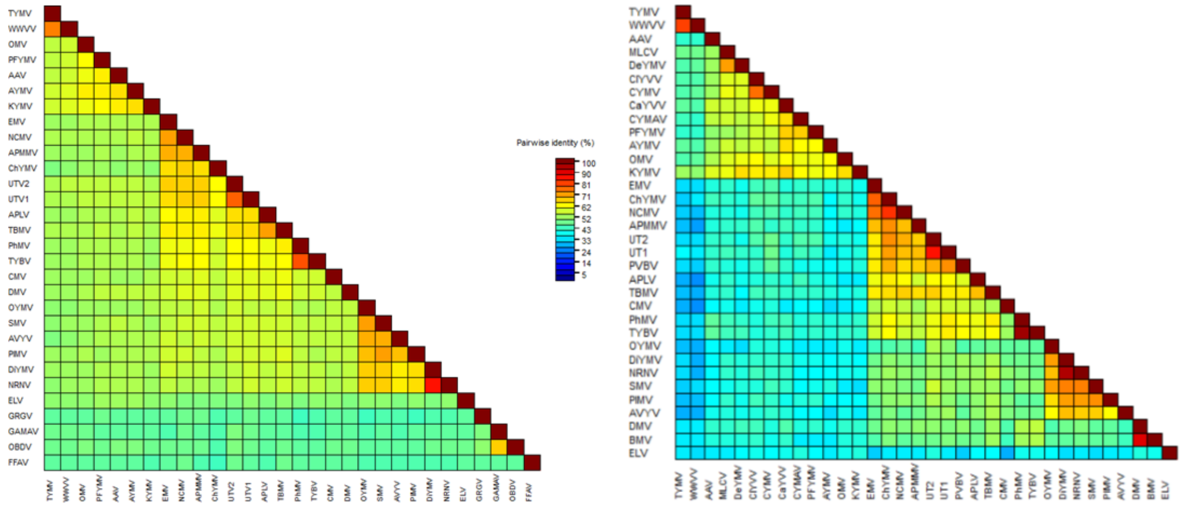

Figure S12. Pairwise matrix for an alignment of tymoviral RPs (left) and CPs (right).
